# Modulating the difficulty of a visual oddball-like task and P3m amplitude

**DOI:** 10.1038/s41598-023-50857-z

**Published:** 2024-01-17

**Authors:** Cindy Boetzel, Heiko I. Stecher, Florian H. Kasten, Christoph S. Herrmann

**Affiliations:** 1 Experimental Psychology Lab, Department of Psychology, European Medical School, Cluster for Excellence “Hearing for All”, Carl von Ossietzky University, Ammerländer Heerstr. 114-118, 26129 Oldenburg, Germany; 2 Neuroimaging Unit, European Medical School, Carl von Ossietzky University, Oldenburg, Germany; 3 Research Center Neurosensory Science, Carl von Ossietzky University, Oldenburg, Germany; 4 Centre de Recherche Cerveau and Cognition, CNRS, Toulouse, France; 5 Université Toulouse III Paul Sabatier, Toulouse, France

**Keywords:** Neuroscience, Psychology

## Abstract

It is often necessary to modulate the difficulty of an experimental task without changing physical stimulus characteristics that are known to modulate event-related potentials. Here, we developed a new, oddball-like visual discrimination task with varying levels of difficulty despite using almost identical visual stimuli. Gabor patches of one orientation served as frequent standard stimuli with 75% probability. Gabor patches with a slightly different orientation served as infrequent target stimuli (25% probability). Analyzing the behavioral outcomes revealed a successful modulation of task difficulty, i.e. the hard condition revealed decreased d' values and longer reaction times for standard stimuli. In addition, we recorded MEG and computed event-related fields in response to the stimuli. In line with our expectation, the amplitude of the P3m was reduced in the hard condition. We localized the sources of the P3m with a focus on those that are modulated by changes in task difficulty. The sources of P3m modulation by difficulty were found primarily in the centro-parietal regions of both hemispheres. Additionally, we found significant differences in source activity between the easy and hard conditions in parts of the pre and post-central gyrus and inferior parietal lobe. Our findings are in line with previous research suggesting that the brain areas responsible for the conventional P3m generators also contribute to a modulation by task difficulty.

## Introduction

Event related neural activity, observed as event-related potentials (ERPs) in the EEG and event-related fields (ERFs) in the MEG, provides an opportunity to investigate the underlying mechanisms of various cognitive processes. Individual components of ERPs appear to reflect the flow of information through the brain. One prominent component that has received considerable research interest is the P3 component (termed P3m in MEG), which can be easily and reliably evoked by the presentation of stimuli in different modalities and is thought to reflect the cognitive processes of stimulus evaluation^1^ and resource allocation^2^.

Typically, the P3 occurs 300–600 ms after stimulus onset and is characterized by a large positive deflection^3^, which’s latency and amplitude can be influenced by different task parameters^4–8^ . It has been shown that different features of the P3 can be altered in several neurological and psychiatric disorders, such as attention deficit hyperactivity disorder^9^, Alzheimer´s disease and dementia^10^, schizophrenia and depression^11^. Therefore, the P3 could serve as a valuable physiological marker for the diagnostic of a wide range of neurological pathologies^12^. If there is indication that the severity of symptoms is related to P3 amplitude and that the amplitude can be influenced by certain medications, as seen in the case of ADHD^13,14^, an alternative approach could involve the attempt to modulate the P3 amplitude without medication. The aim would be to bring it to a "normalized" level and potentially alleviate symptom severity. One conceivable approach would be an electrical stimulation of the brain activity using non-invasive brain stimulation (NIBS) methods. Here the brain oscillations, which are presumably involved in the generation of the ERP^15^ (for a critical discussion, see^16^) and thus also the P3 amplitude, could be modulated with transcranial alternating current stimulation (tACS). Before attempting to modulate P3 amplitude with tACS, it is essential to explore the origins of the P3, as well as the sites where the P3 modulation occurs due to pathological conditions or changes in task parameters.

The origin of the P3 in the brain has been the subject of several studies. Several different source locations have been reported for the visual P3 component. For example, Rogers and colleagues^17^ identified two simultaneous but spatially distinct P3 sources. One in deep right hemisphere structures below the temporal cortex, close to the right hippocampal formation, and the second one in the primary visual cortex^17^. Okada and colleagues^18^ located the source of the visual N2-P3 complex in the hippocampal formation. Using MEG, Mecklinger and colleagues^19^ identified neural generators of the P3m in subcortical structures close to the thalamus. In addition to these sources, the superior and inferior frontal lobes, the middle temporal gyrus, the parietal lobe and the cingulate gyrus were shown to be prominent sources of the P3m^20^. These findings provide evidence for a widespread network involved in the P3/P3m generation.

Although the origin of the P3 itself is well investigated, it remains unclear whether the P3 modulation that occurs due to pathological conditions or changes in task parameters, such as task difficulty, arise from the same brain regions as the conventional generators of the P3m. A precise identification of the origins of P3m and the corresponding modulation of P3m due to task difficulty within the specific modality and task is essential for NIBS. This is because, for such stimulation, a certain degree of focality is required to increase the stimulation effects. Imprecisely defined target regions could result in minimal or no effect, as the source(s) may not be adequately stimulated.

In order to identify the brain regions, where the modulation by task difficulty of the P3m originates, it is essential to employ a task that induces a P3m amplitude modulation while preserving the target/standard effect that is typically observable in oddball-paradigms. The target/standard effect refers to the variation in P3(m) amplitude in response to infrequent trials (referred to as targets) as compared to frequent trials (referred to as standards). In so-called oddball-paradigms, targets are presented at probabilities of e.g. 20 or 25% while the standard stimuli are presented at probabilities of e.g. 80 or 75%, respectively. Such paradigms, have been shown to reliably elicit a higher P3 amplitude for targets as compared to standards. This effect can be influenced by factors such as stimulus probability^7^ and inter-stimulus interval (ISI)^6^, with the difference diminishing for higher target probabilities and smaller ISIs. Another task parameter, that can be modulated is task difficulty. Previous studies indicate that increased task difficulty results in a reduced P3(m) amplitude^4–6,21^, although the underlying mechanisms of the amplitude modulation are not totally clear, as task difficulty can be varied extensively. It is important to note that different changes to the task may not have the same effect on the underlying neuronal processes required to complete the task. For instance, varying physical stimulus parameters such as size, color, contrast and shape impact not only early ERP components^22,23^ but also the P3 amplitude^24^. Differentiating between e.g., smaller differences in contrast or shape, involves distinct neuronal processes compared to task modulations with a different stimulus ratio. Changes in physical parameters induce an amplitude modulation for both targets and standards, but not the difference between them (target/standard effect). The difference is modulated when the probability of targets is changed (e.g., from 25 to 10%), altering the amplitude of the target P3 in relation to the standard P3 and thus modulating the target/standard effect.

In general, attributing the modulation of the P3 to either distinct physical properties or the difficulty of the task becomes challenging when only examining electrophysiological outcomes, such as P3(m) amplitude. Therefore, behavioral outcomes must be included in the analysis and the interpretation of the data.

In this study, our aim was to establish a visual task that can be used to reveal the site of the P3m modulation dependent on task difficulty. This required a task that is capable of evoking modulated ERFs without interfering with the target/standard effect. This was a necessary condition for demonstrating the feasibility of using tACS to modulate the P3m amplitude in a proof-of-concept context in a follow-up study^25^. To achieve this, we developed and examined a visual task paradigm in MEG with two different levels of task difficulty, categorized as easy and hard. We used Gabor patches to maintain that physical parameters of the stimuli constant known to modulate ERP components, such as spatial frequency^23^, color, brightness and stimulus size^22,24^. By adjusting the rotation of the sine-wave gratings (away from vertical), we increased the task difficulty while preserving all other physical properties of the stimuli^26^. Consequently, the modulation of the P3m amplitude can clearly be attributed to changes in task difficulty achieved by different rotations.

We expected that by increasing the task difficulty, there would be a decrease in P3m amplitude in the hard condition compared to the easy condition. In addition, we hypothesize a reduced performance level (d’–d prime) and increased reaction times in the hard condition as compared to the easy condition. The behavioral outcomes are investigated to prove the increased task difficulty. Subsequently, we examined the origins of the task difficulty-dependent P3m modulation by employing a Linearly Constrained Minimum Variance (LCMV) beamformer. This investigation aimed to pinpoint the target region for a subsequent study, recently published, where ERP-aligned tACS was administered to modulate P3 amplitude in healthy participants^25^. In addition, if an experimental task can be established in which difficulty can be easily adapted in arbitrary degrees, this also allows to use the task in multiple subjects’ groups with different abilities. This is often required when transferring an experiment from young to elderly subjects or when comparing patients to a healthy control group.

## Methods & material

### Participants

The study included 19 participants (12 females, mean age 25.4 years, ranging from 18 to 31 years) with no self-reported history of psychiatric or neurological disorders. Exclusion criteria such as the presence of metallic materials in the head and a family history of epilepsy (first-degree relative) were obtained before the start of the experiments. Other inclusion criteria were normal or corrected-to-normal vision, right-handedness, and non-smoking. One subject was excluded because of incorrect segmentation of the MRI scan, making source analysis impossible. Two further participants had to be excluded because their d’ in the easy condition was less than 1.5 times the standard deviation of the mean d’ values in that condition. One subject fell asleep while performing the tasks. This left 15 participants (9 females) for data analysis. All participants had to participate in a single MEG session, which consisted of a short training session at the beginning to familiarize them with the task. Task difficulty was manipulated between conditions, with the order of the conditions randomized and counterbalanced across participants. Ethical approval for the study was granted by the “Kommission für Forschungsfolgenabschätzung und Ethik” (“*Commission for Research Impact Assessment and Ethics*”), the institutional review board at the University of Oldenburg for non-medical studies (Drs. Nr. EK/2019/036). All experimental protocols and methods were performed in accordance with relevant guidelines and regulations. Informed consent was obtained from all participants.

### Visual task

**Figure 1:**
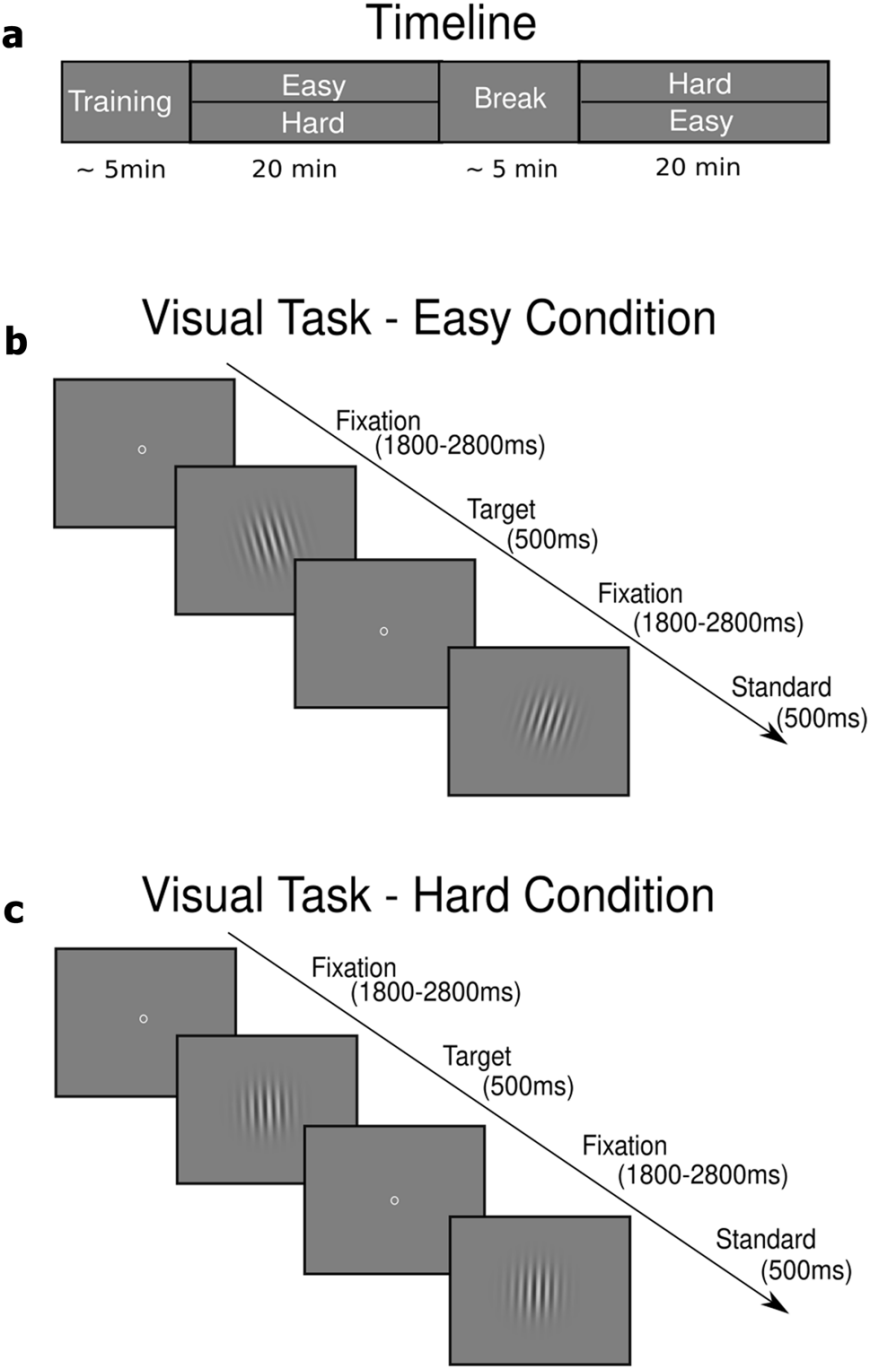
Experimental setup. **(a)** Timeline of the experiment. The experiment started with a short training session, followed by either the easy or the hard condition. After a short break, the participants had to perform the second experimental condition. Each experimental condition lasted 20 min. **(b)** Visual task in the easy condition. Centric circles served as fixation. The interstimulus interval jittered between 1800 and 2800 ms. Visual stimuli appeared for 500 ms followed by an inter-stimulus interval. The rotation angle of the Gabor patch was 2°. **(c)** Visual task in the hard condition. The rotation angle of the Gabor patch was 0.5°. For visualization, the rotation angles were increased compared to the experimental rotation angles.

To evoke the ERFs, participants had to perform a visual, oddball-like task. The time course of the experiment is shown in Fig. 1a. The visual task is shown in Fig. 1b, c. The Gabor patches, generated in Matlab R2019a (The MathWorks Inc., Natick, MA, United States), were rotated either to the left or to the right and had 30 pixels per cycle (resulting in a spatial frequency of 3.8 cycles per visual degree angle). The Gabor stimuli were presented using a custom-made presentation script (Presentation version 18.1, Neurobehavioral Systems Inc.). The distance between the participants and the monitor was kept constant in order to keep the perception of the stimuli constant for each participant. At the beginning of each trial, a small circle appeared in the center of the screen as a fixation point. After a randomized interval of 1800 to 2800 ms, the Gabor patch appeared in the center of the screen for 500 ms. Participants had to respond between 200 and 1300 ms after stimulus onset and were instructed to press the button as quickly and correctly as possible. The fastest reaction time considered a correct response was set at 200 ms after stimulus onset, as faster responses cannot be considered a response to the stimulus due to limitations in neural transmission speed^27^. Participants had to respond to each stimulus by pressing a corresponding button (left or right) with their index finger, which differed from the normal oddball paradigm, in which participants only respond to the infrequent stimuli. Each condition consisted of 400 trials (100 target and 300 standard trials) and lasted approximately 20 min. To reduce potential confounds due to the orientation of the standard and target stimuli, participants were assigned to one of two target orientation groups. That is, if a participant was in group one, the target stimulus was rotated to the left and the standard stimulus was rotated to the right. In group two, the assignment was reversed. The rotation direction of the target stimuli was counterbalanced across subjects (left-rotated: 8 participants). Target stimuli were presented with a 25% probability, while standard stimuli had a 75% probability. This specific ratio has been demonstrated in previous studies to effectively elicit differences in P3 amplitude (target/standard effect) in both EEG^28^ and MEG^29^, serving as evidence that our paradigm was functioning as intended, with the target/standard effect remaining unaffected. This rather high target probability was chosen to ensure a sufficient number of trials for a robust ERF, while still representing an infrequent oddball-like event that should produce an increased P3m compared to standard trials. To ensure that the target stimuli were perceived as oddballs, some constraints on stimulus presentation were introduced. Each condition began with a sequence of at least 10 standard stimuli. Throughout the experiment, a maximum of two targets could appear in succession. The difficulty of the task was regulated by the rotation. Each participant performed a short practice with three different conditions of increasing difficulty (easy: 2°; medium: 1° and hard: 0.5° from the vertical line) to familiarize them with the task. Each practice condition consisted of 15 trials, including 4 target trials. Each participant then performed each condition (easy and hard conditions) of the visual task.

### Magnetoencephalogram

During both experimental sessions, neuromagnetic signals were recorded using a 306-channel whole-head MEG with 102 magnetometers and 204 orthogonal planar gradiometer sensors (Elekta Neuromag Triux System, Elekta Oy, Helsinki, Finland). In preparation for each MEG measurement, 5 head-position indicator (HPI) coils were attached to the head of each participant—three coils at the frontal hairline and one coil at each mastoid, respectively. The position of the coils was recorded together with anatomical landmarks (left and right tragus and nasion) and at least 200 samples of the head shape using a Polhemus Fastrak (Polhemus, Colchester, VT, USA). During the MEG measurements, the sensor array was in an upright position (60° dewar orientation) and the data were sampled at 1000 Hz.

### MRI acquisition

To perform source analysis of the individual P3m modulations, a structural MRI was obtained from each subject. The MRI scans were acquired using a Siemens Magnetom Prisma 3 T whole-body MRI scanner (Siemens, Erlangen, Germany). A T1-weighted 3-D sequence (MPRAGE, TR = 2000 ms, TE = 2.07 ms) with a slice thickness of 0.75 mm was used.

### Data analysis

Data analysis was performed in Matlab R2019a using the fieldtrip toolbox for MEG processing^30^. External noise present in the MEG was suppressed using the time–space signal separation (tSSS) method with default settings (*L*_in_ = 8, *L*_out_ = 3, correlation limit = 0.98^31,32^) using MaxFilter v2.2 (Elekta Neuromag, Elekta Oy, Finland). Raw data were resampled to 250 Hz. The data were filtered between 1 and 30 Hz, detrended and cut into 5 s epochs. The epochs were then used for an ICA approach to remove components containing horizontal and vertical eye-movements and heartbeats (mean: 9 components rejected, SD: 3 components) before the data were back-projected into sensor space. The cleaned data was then filtered between 0.1 and 13 Hz using a 4th-order Butterworth filter. The low pass filter was set at 13 Hz to emphasize the P3m amplitude, whose frequency lies between the delta (0.5–3.5 Hz) and theta (~ 4 to 7.5 Hz^33^) bands but still including the high alpha frequency band. To extract the ERF, the data were cut into epochs of -0.2 to 1 s around the stimulus onset. A baseline correction (−0.2 to 0 s) was applied and the individual epochs were averaged. Only correctly responded trials were included in the analysis. This yielded 90 trials for the easy targets and a standard deviation (SD) of 8 trials, along with 76 trials involving hard targets and a SD of 23 trials. Additionally, there were 285 trials for the easy standard trials with a SD of 32 trials, and 197 trials for hard standards with a SD of 56 trials available for analysis.

To examine behavioral changes between the easy and hard conditions, d’ and RTs were calculated for each participant and for each condition. D’ was calculated by subtracting the z-transformed false alarms rate from the z-transformed hit rate.

### Statistical analysis

Statistical analyses were performed in Matlab R2019a using the fieldtrip toolbox. For statistical analyses, only the gradiometer signals combined over both directions were considered, to avoid signal cancellation. To investigate, how task difficulty affected the P3m amplitude, the ERFs recorded by the combined gradiometers were averaged for each participant. Combined gradiometers were created by calculating the magnitude of the planar gradient in both directions, which combines the two gradients at each sensor into a single positive-valued number. The ERFs were first combined for each participant and then averaged. To investigate the effect of the difficulty modulation, the combined ERFs of each participant’s gradiometers was used for a one-tailed dependent samples cluster-based permutation test (CBPT) with 10,000 random draws^34^ and Monte Carlo estimates to calculate the p-values for data between −0.2 and 1 s after stimulus onset. The reason for employing a CBPT test for analyzing the task-difficulty effect statistically, as opposed to conventional methods like ANOVAs or similar approaches, is the CBPT´s ability to identify spatial or temporal patterns in the data without presupposing the specific location or time interval of the anticipated effect. The CBPT reveals, if present, significant clusters in the data, while considering the temporal and spatial information. Furthermore, the CBPT inherently accounts for multiple comparisons within identified clusters, thereby providing a more precise assessment of statistical significance. Time intervals exhibiting significant clusters were defined as times of interest (TOI), while channels displaying significant clusters were defined as regions of interest (ROI). One CBPT was performed for each stimulus type (targets and standards) and for the average of both stimulus types (combined). These assignments were utilized for visualizing the results. In order to examine the target/standard effect, wherein lower P3m amplitudes for standards are anticipated, we extracted the ten channels with the highest amplitude between 0.4 and 0.6 s (as indicated by the grey bar in Fig. 4) following stimulus onset in the easy target condition. The ten channels were selected based on the easy target condition, given the anticipation of the highest P3m amplitude in that particular condition. The time interval was selected, as the peak of the P3m amplitude in that condition was at 501 ms after stimulus onset. Subsequently, the amplitude values of the ten channels and the chosen time interval were averaged for each participant and each condition/stimulus type, respectively. The target/standard effect was then assessed using a repeated measures ANOVA (rmANOVA) with a 2-level factor condition (easy/hard) and a 2-level factor stimulus (target / standard).

The TOI of each stimulus type from each CBPT was used as the time-interval for the LCMV beamformer to localize the sources of the significant difference between the easy and hard conditions. Therefore, the difference between the easy and hard conditions during the TOI was calculated by subtracting the power values of the hard condition from those of the easy condition for each stimulus, respectively (∆ targets; ∆ standards). A further CBPT with 1000 random draws^34^ and Monte Carlo estimates was then computed to test the difference between the easy and hard conditions at the source level.

The task difficulty-dependent difference in d’ was investigated using a two-sided t-test, while the effect of the changes in task-difficulty on the RTs was assessed using a rmANOVA with a 2-level factor condition (easy/hard) and a 2-level factor stimulus (target/standard). Post-hoc analyses for untangling interaction effects were carried out using two-sided t-tests with Bonferroni correction.

### Linearly constrained minimum variance (LCMV) beamformer

The P3m amplitude modulation induced by task difficulty was projected into source-space using an LCMV beamformer^35^. To do this, the ERFs of the individual participants were first calculated (from −0.2 to 1 s around stimulus onset). These ERF data were projected onto a 6 mm grid and warped into MNI (Montreal Neurologic Institute) space. A single-shell head model for each participant was co-registered with the participant´s head position in the MEG. The combined ERF data of the target and standard trials were used to compute a common filter, which was subsequently applied to the projection of the target and standard data, respectively. As we investigated the effect of task difficulty on each stimulus type separately, different TOI extracted from the CBPT of the ERFs were used to compute the P3m sources. These source data were averaged across participants for each stimulus type. To investigate the location of the task difficulty-dependent P3m modulation, we calculated the difference of each stimulus type between the hard and the easy conditions (hereafter referred to as ∆ combined, ∆ targets and ∆ standards). In addition, a one-tailed correlation cluster test with 1000 randomizations was performed to test for a correlational relationship between the performance difference and the source activation difference between the easy and hard conditions. Cluster p-values were corrected for multiple comparisons.

## Results

### Behavioral data

**Figure 2:**
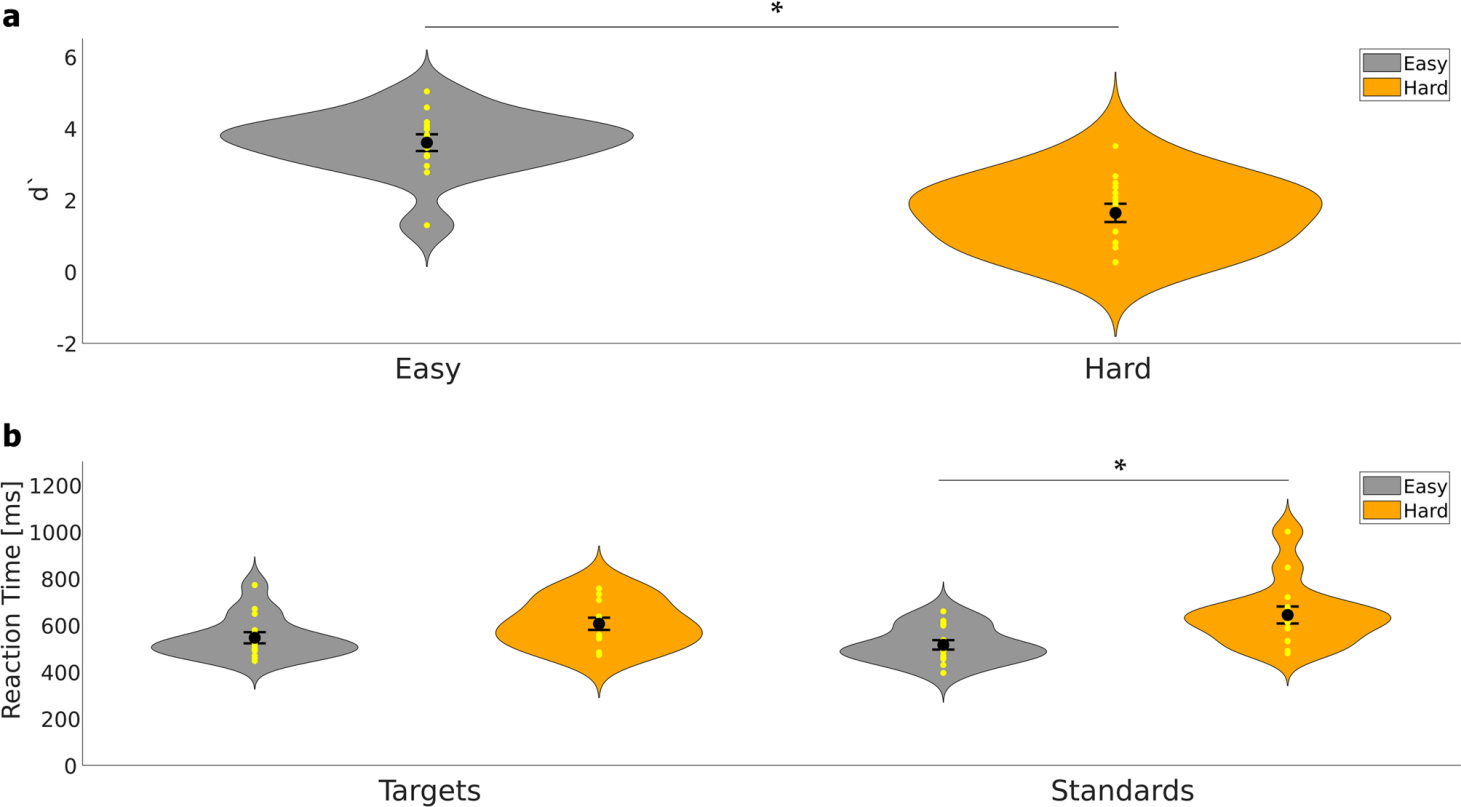
Behavioral data. **(a)** D’ of the oddball-like visual discrimination task. Yellow dots indicate single data points. Big black dots represent the mean of the data and error bars indicate the standard error of the mean. Grey violins represent the easy condition and orange violins the hard condition. The violins of d’ hardly show an overlap, which means that there is a clear difference in the performance between the easy and hard conditions, which is supports the statistical analysis of the data. (**b**) RTs for targets and standards, respectively. RTs increase in the hard condition compared to the easy condition, but a significant difference is observed only for standards.

To verify that our manipulation of task difficulty in the newly developed experimental paradigm worked, we tested for changes in performance and RTs. Figure 2a shows the d’ for the easy and hard condition and Fig. 2b shows the RTs for targets and standards in each condition, respectively. The d’ value differed significantly in the hard condition as compared to the easy condition (t_(28)_ = 5.866, p < 0.01, Cohen´s d = 2.142). These results confirm a considerable performance decrease achieved by increasing the task difficulty.

The rmANOVA for the RTs showed a significant main effect for *condition* (F_1,14_ = 15.111, p < 0.01, η^2^ = 0.519) and a significant interaction *condition x stimulus* (F_1,14_ = 18.627, p < 0.01, η^2^ = 0.571). The main effect *stimulus* did not reach significance (F_1,14_ = 0.041, p = 0.842, η^2^ = 0.003). To disentangle the significant interaction effect, we performed two-sided t-tests for: easy vs. hard targets; easy vs. hard standards, target vs. standard easy trials, and target vs. standard hard trials. For easy vs. hard targets, the t-test did not show a significant difference (t_(28)_ = −1.747, p = 0.160, Cohen’s d = −0.638), while for easy vs. hard standards, the test showed a significant effect (t_(28)_ = −3.186, p = 0.014, Cohen’s d = −1.163; Bonferroni-corrected for four comparisons) indicating an increase in RTs for standard trials in the hard condition. There was neither a significant difference in RTs for target vs. standard easy trials (t_(28)_ = 1.008, p = 0.322, Cohen’s d = 0.368) nor for target vs. standard hard trials (t_(28)_ = −0.870, p = 0.391, Cohen’s d = −0.318).

### P3m amplitude modulation induced by task difficulty

Next, we tested whether the differences in task difficulty observed at the behavioral level were reflected in the P3m amplitude at the sensor level. The CBPTs showed a difference between the easy and hard condition for each stimulus type.

**Figure 3:**
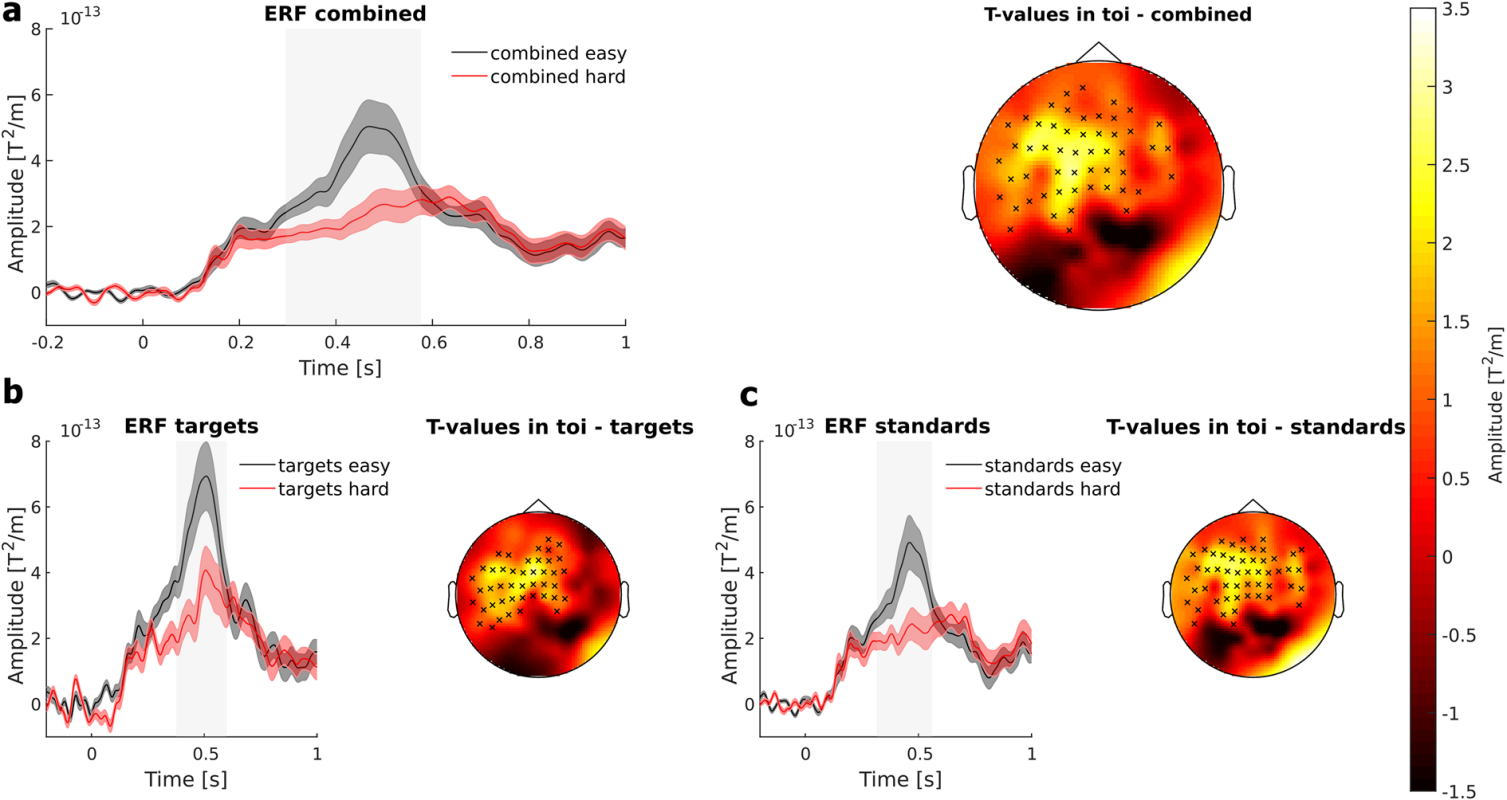
Visualization of cluster-based permutation test of difficulty effect. **(a)** Combined ERFs of gradiometers revealing a significant difference between the easy and hard condition (indicated by small crosses in topography top right). The black line indicates the easy condition, red line indicates the hard condition. Shaded error bar represents the standard error of the mean. Grey bars indicate TOI. The topography (top right) shows the t-values resulted from the CBPT averaged over the TOI. Black crosses indicate the channel, associated with the significant cluster. (**b**) ERFs and topography with t-values for target trials. **c** ERFs and topography with t-values for standard trials.

For targets and standards combined, the CBPT revealed a significant cluster ranging from approximately 296 to 576 ms after stimulus onset (p < 0.01). For targets only, a separate CBPT revealed a significant cluster ranging from approximately 376 to 600 ms (p = 0.026) after stimulus onset. Similarly, when examining only standards, another CBPT identified a significant cluster spanning from about 316 to 560 ms (p < 0.01) after the stimulus onset. Time intervals with significant clusters were defined as times of interest (TOI) for further visualization steps. To visualize the differences between the easy and hard conditions for each stimulus type in the time-domain, the channels forming the significant clusters (see Fig. 3, small black crosses in the topographies) were averaged and the resulting signal traces are shown in Fig. 3 as ERFs. Note, that each visualized ERF comprises slightly different channels, as each CBPT (for combined, target and standard trials, respectively) has identified distinct channels to be included in the significant cluster. The ERFs clearly showed a reduced P3 amplitude in the hard condition (red line) as compared to the easy condition (black line) for each stimulus type (Fig. 3). The topographies showed a fronto-central distribution of the differences between the easy and hard conditions, indicating that these fronto-central regions are involved in P3m amplitude modulation. To further investigate the sources of the task difficulty dependent P3m modulation and to test whether the P3m modulation is located in fronto-central regions, the averaged ERFs of each stimulus type (targets and standards) were subjected to a source localization analysis using LCMV beamforming (Fig. 5).

**Figure 4:**
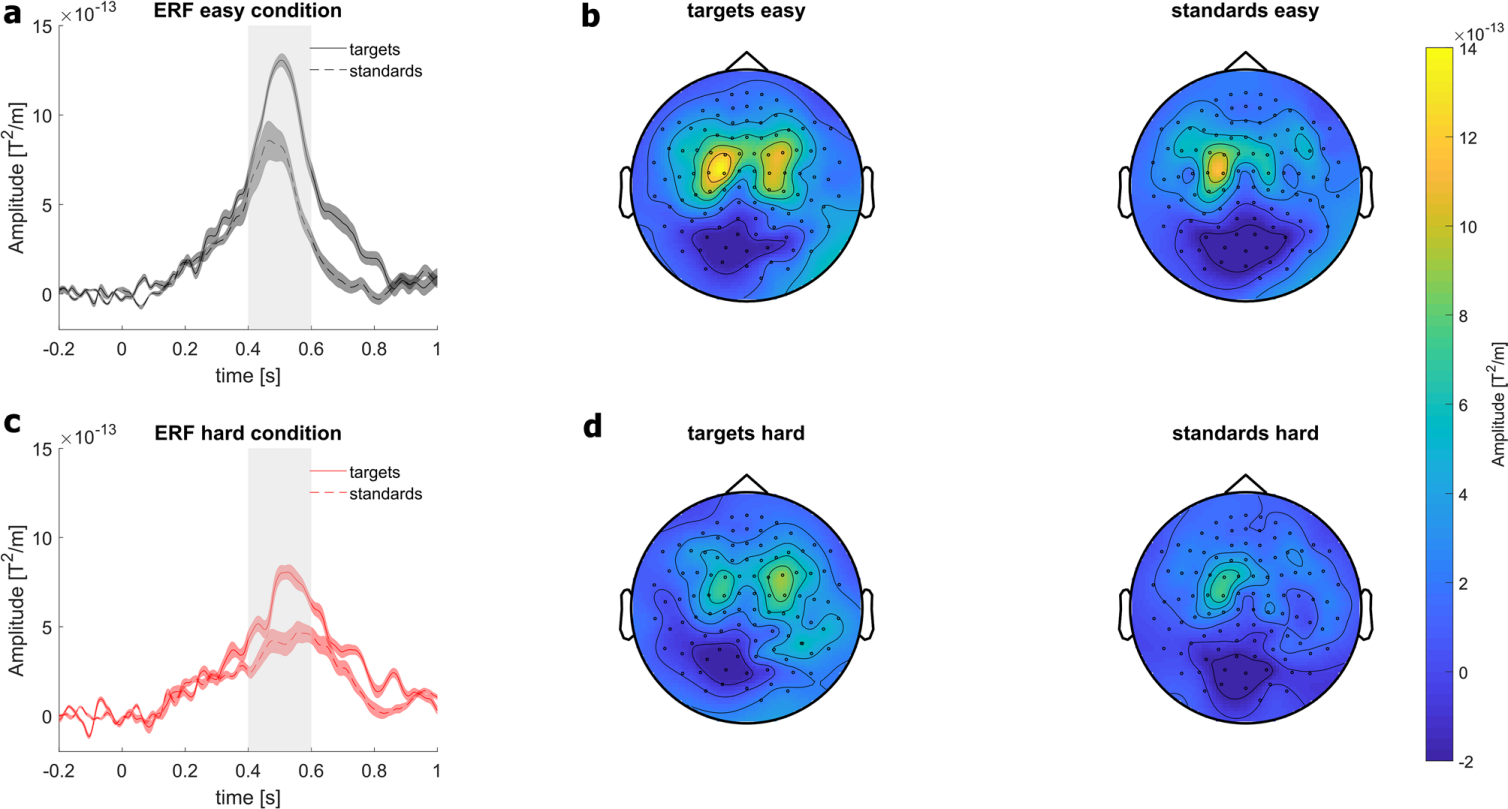
Visualization of target/standard effect. (**a**) ERFs in 10 channels with the highest amplitude between 0.4 and 0.6 s (indicated by the grey bar) following stimulus onset. The 10 channels were selected from the easy target condition. The shaded error bar represents the standard error of the mean. Solid lines indicate the ERFs of targets and the dotted lines indicate the ERFs of standards. (**b**) Topographies of combined gradiometer for targets and standards in the easy condition in the time interval between 0.4 and 0.6 s. respectively. (**c**) ERFs in the hard condition for targets and standards, respectively. (**d**) Topographies of combined gradiometer for targets and standards in the hard condition in the time interval between 0.4 s and 0.6 s. respectively.

In a secondary analysis, we examined the target/standard effect produced by the oddball-like paradigm (Fig. 4). As the P3m is typically elicited in an 'oddball' paradigm when a participant detects an infrequent 'target' stimulus in a series of consecutive standard stimuli^36^, we expected a reduced P3m amplitude in standard trials compared to target trials, although this was not essential for the task difficulty-dependent modulation effect. The rmANOVA revealed a significant main effect for *condition* (F_1,14_ = 13.057, p < 0.01, η^2^ = 0.483) and *stimulus* (F_1,14_ = 8.214, p = 0.012, η^2^ = 0.370; grey area in Fig. 4), but not for the interaction *condition x stimulus* (F_1,14_ = 0.921, p = 0.354, η^2^ = 0.062). The target/standard effect on source level is shown in Supplementary Fig. 1.

### Sources of the task difficulty-dependent P3m modulation

To validate the localized sources of the P3m, we also examined the source activity for the P1m (90–110 ms after stimulus onset) and the N1m (160–180 ms after stimulus onset). The results are shown in the Supplementary Material (Supplementary Fig. 2, 3, 4) for combined, targets and standards, respectively. As expected, early activation for the P1m was located in occipital regions. For the N1m, the highest activity was located in the lateral occipital complex.

**Figure 5:**
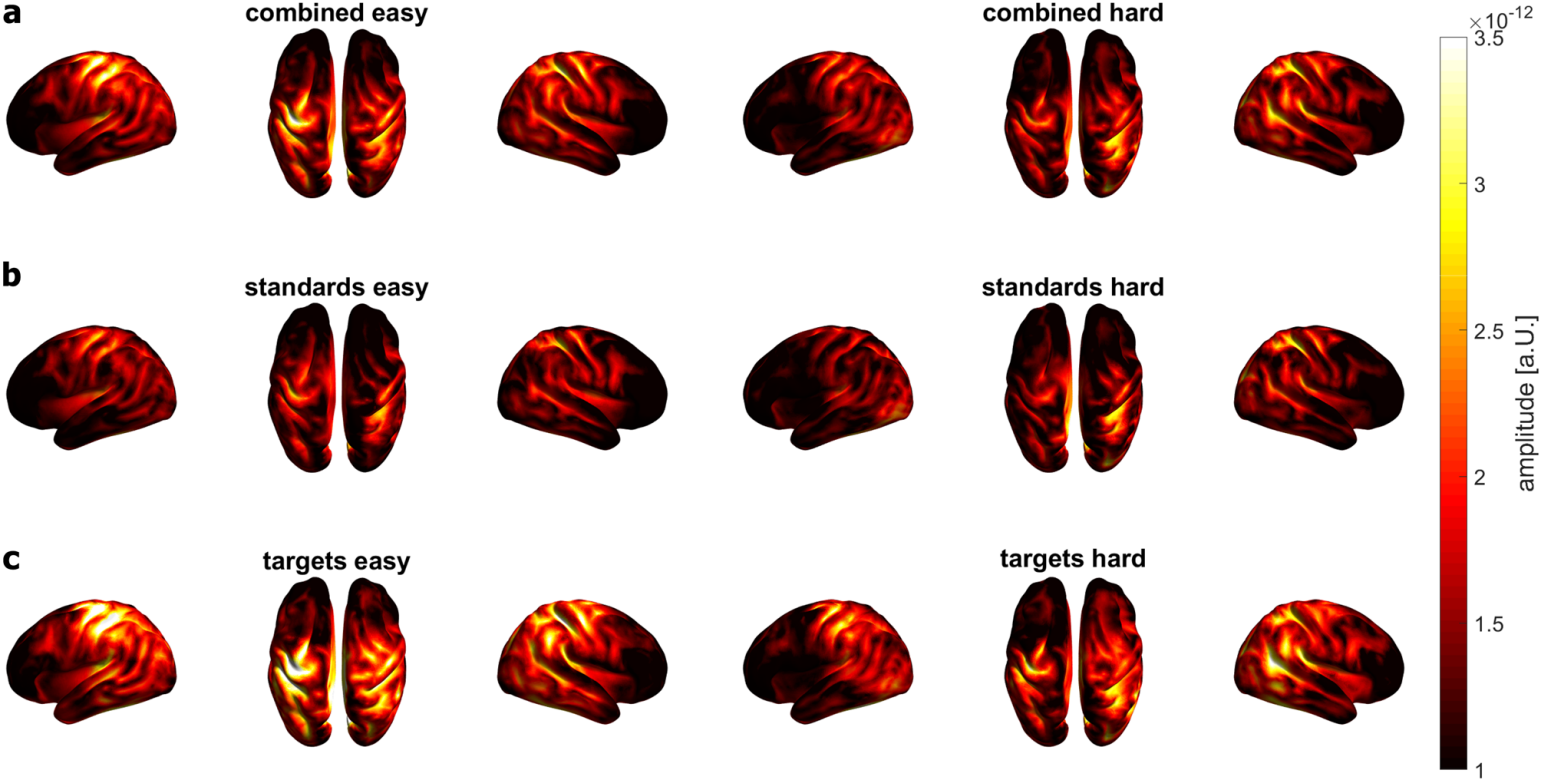
Averaged results of the LCMV beamformer for the TOI (P3m) for each stimulus type and each condition. **(a)** Source activity for combined stimuli for the easy (left) and hard (right) condition. Absolute values of source activity are depicted. The highest activity for combined stimuli in the easy as well as in the hard condition is in the centro-parietal and temporal regions. (**b**) Source activity for standard stimuli. For standard stimuli, the source activity in the centro-parietal and temporal regions is lower as compared to the source activity of combined stimuli. (**c**) Source activity for target stimuli. For target stimuli, the source activity is much more pronounced in the centro-parietal and temporal regions as compared to the source activity of standard stimuli. In addition, the middle occipital gyrus of the left hemisphere shows a high source activation.

**Figure 6:**
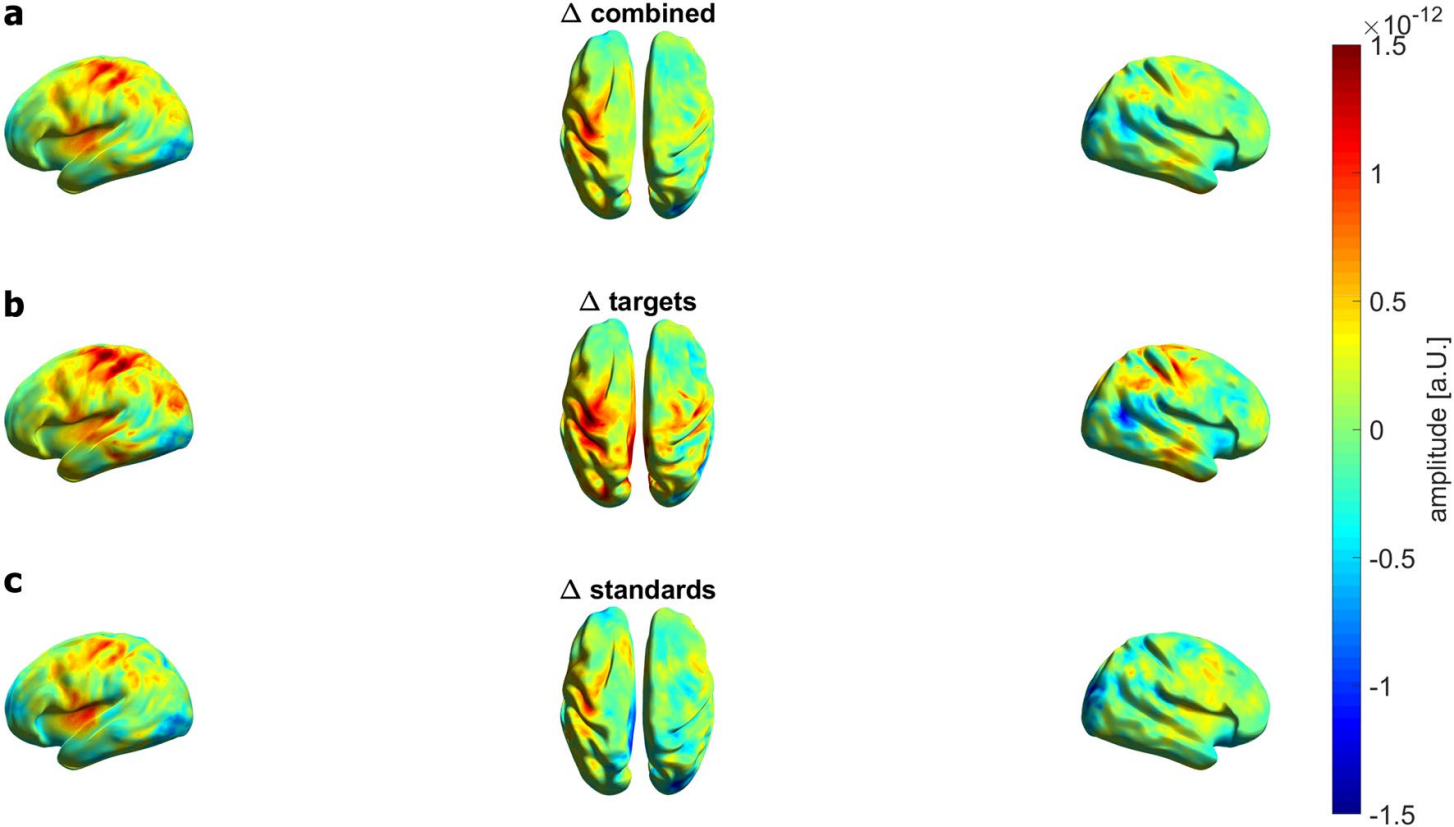
Grand averaged results of the LCMV beamformer ∆ combined, ∆ targets and ∆ standards. (**a**) Source activity for ∆ combined, with the highest source activity in centro-parietal and temporal regions. Difference of the absolute values of source activity are shown. (**b**) Source activity for ∆ targets with a more pronounced activity in centro-parietal and temporal regions of both left and right hemispheres. (**c**) Source activity for ∆ standards. For the standards, as compared to the targets, the source activity is mainly located in the insula and centro-parietal regions of the left hemisphere.

The following section focuses on the results of the P3m. The averaged results of the LCMV beamformer are shown in Fig. 5. The highest source activity for combined stimuli in both easy and hard conditions was in the centro-parietal and temporal regions. For the standard stimuli, the source activity was weaker as compared to the source activity of the combined stimuli. For the target stimuli, the centro-parietal source activity was much more pronounced than for the combined stimuli. To investigate the location of the task difficulty-dependent P3m modulation, we first calculated the differences in source activity between the easy and hard conditions (Fig. 6) to visualize the task difficulty-dependent activation modulation descriptively. The most prominent source activity for the activity difference across all stimulus conditions was located in centro- and posterior parietal regions of the left hemisphere. Target and standard stimulus activity patterns showed clear differences.

The activity difference between easy and hard targets showed a more pronounced activity in centro-parietal and temporal regions, and the insula of both left and right hemispheres. The middle occipital gyrus of the left hemisphere seemed to show a higher source activation in the hard condition compared to the easy condition, as the difference plot (Fig. 6) shows negative values in this region.

**Figure 7:**
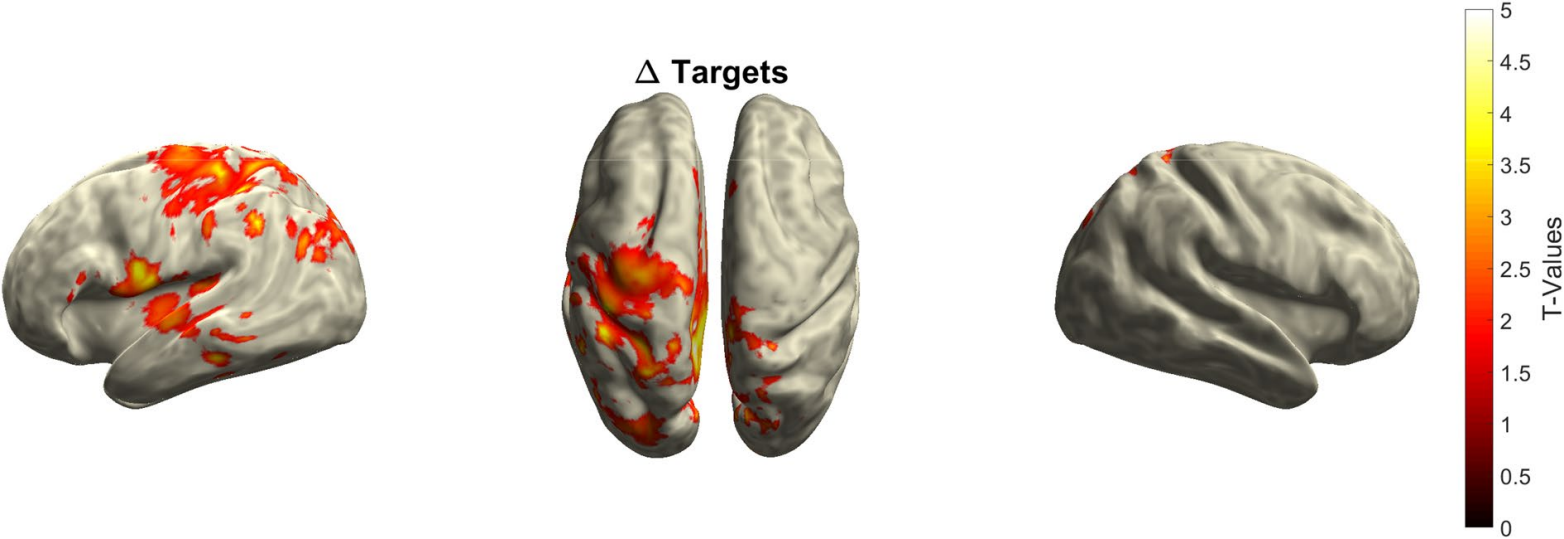
Results of the CBPT on source level. Only the CBPT for target trials revealed a significant difference between the easy and hard condition during the TOI (p < 0.01). The significant difference for target trials is mainly located in the insula and centro-parietal and temporal regions of the left hemisphere. The color bar shows the t-values of the CBPT.

We further investigated the difference between the easy and hard conditions statistically using a CBPT for each stimulus type, respectively. The CBPT revealed a significant cluster for the target trials (p_Cluster_ < 0.01; see Fig. 7), but not for combined (p_Cluster_ = 0.550) and standard trials (p_Cluster_ = 0.563).

The significant difference between the easy and hard conditions for target trials was mainly located in centro-parietal and temporal regions, and the insula of the left hemispheres. There was no correlation between performance and source activity modulation for either target trials (p_Cluster_ = 0.893) or standard trials (p_Cluster_ = 0.793). In summary, at the source level, there was significant task difficulty modulation only in target trials, and the modulation was primarily located in the left hemisphere.

## Discussion

In this study, our aim was to establish a visual task capable of reliably evoking ERFs while modulating the difficulty and, in turn, the amplitude of the P3(m). Furthermore, we examined the origins of the task difficulty-dependent P3m modulation by employing a LCMV beamformer. A precise localization of the task difficulty-dependent P3m modulation would be essential for further studies, investigating the feasibility of tACS to modulate the P3(m).

To this end, we developed a visual task paradigm, that allowed us to increase the task difficulty while keeping the physical properties of the stimuli mostly constant, a unique feature compared to previous studies investigating the task difficulty-dependent modulation of the P3. With the established visual task, we obtained a reduced level of performance and significantly increased RTs in the hard condition for standards and descriptively increased RTs in the hard condition for target trials. Furthermore, a reduced P3m amplitude was found in the hard condition compared to the easy condition. The source activity associated with the task-difficulty dependent P3m modulation in target trials was primarily located in centro- and posterior parietal regions in both hemispheres. The pattern of the source activity for standard trials showed a descriptively similar pattern as to target trials. In addition, parts of the pre- and post-central gyrus and the inferior parietal lobe showed a significant difference in source activity (see Fig. 7) for target stimuli. A correlation cluster test did not uncover any correlation between performance and the modulation of source activation.

At the sensor level, previous studies have demonstrated the effect of different task difficulties on P3 amplitude^4,5,8^, with higher task difficulties reducing P3 amplitude. These findings support the results of the present study, as the hard condition evoked a significantly lower P3m amplitude as compared to the easy condition (see Fig. 3). One explanation for these findings could be that the P3m is associated with memory demands and attentional processes^21,37,38^. In the easy condition, the physical difference between the two stimulus types was designed to be so obvious that only a small amount of attention was required to distinguish the target from the standard stimulus. In addition, uncertainty about the stimulus category is accompanied by a decrease in P3m^3,8,21^. In the present study, the participants seemed to be sure whether the stimulus was rotated to the left or to the right. This is reflected in high performance level and low RTs in the easy task (see Fig. 2). However, as the difficulty of the task increased, the difference between left- and right-rotated stimuli was rather small. Presumably, adjusting the task difficulty in our study led to a lower discriminability of the physical parameters of the stimuli, so that participants were less sure about the stimulus category. This assumption is supported by the rather large decrease in the performance level and the increased RTs (see Fig. 2). If participants had been certain about the stimulus and the correct category classification, but had had to allocate more attentional resources to perform the task, the P3m amplitude would have been increased^3,21^. Therefore, it is reasonable to assume that participants in the hard condition had more problems classifying the stimuli into the correct category, rather than using more resources to perform the task.

Other parameters that can influence the amplitude of the P3 are the ISI, the probability of the stimuli, and the relevance of the stimulus to the task^3^. In this study, all these parameters were maintained similar across conditions to eliminate the possibility of these additional factors being a cause for variations in the P3m amplitude and to ensure that the target/standard effect is unaffected. Thus, we are confident that the P3m modulation was solely due to the changes in task difficulty, as confirmed by the behavioral outcomes and the missing interaction between *condition x stimulus* in the rmANOVA testing for the target/standard effect.

The ERF-findings are supported by the behavioral data, which showed a significantly reduced d' in the hard condition as compared to the easy condition, confirming our experimental manipulation. In addition to a reduced level of performance, our data showed increased RTs for both stimulus types in the hard condition. However, only the RT increase for standards was significant.

There were some discrepancies between our expectations and the measured data. First, we expected an increase in RTs for both target and standard stimuli. It should be noted that the probability of a target stimulus appearing is much lower (25%) than that of a standard stimulus (75%). Therefore, based on the number of trials alone, the increase in RTs is likely to be estimated more accurately for the high number of standards than for to the low number of targets. It may also be possible that the change in rotation direction from a standard stimulus to a target stimulus was more salient than the repetition of successive standard trials where the rotation direction remained the same, and therefore the RTs did not increase for targets, as the targets may appear to ‘pop-out’.

Our behavioral results (d’) are in line with the P3m modulation on sensor level, as they show significantly reduced P3m amplitudes for target and standard stimuli. This finding reflects the relation between the performance-level and the P3m amplitude as previously described in several studies^4,5,8^. However, we were able to show that the P3m modulation also occurred in a paradigm in which all other physical properties, except the rotation direction of the Gabor patch, were maintained constant. Thus, we can exclude that the modulation of the P3m amplitude was achieved by physical differences, for example, the size or the contrast of the stimulus, since such parameters have been shown to modulate the P3m amplitude by themselves^24^.

Following up on the physiological results at the sensor level, we expected a reduced source activation for the combined stimuli as well as for each stimulus separately, but only the source activation for the targets decreased significantly. An explanation might be that the brain activity in target trials is much higher as compared to the standard trials (see ERFs in Fig. 3 and source activation in Fig. 5). Therefore, there is a higher potential for brain activity to be reduced by task modulation in the target trials compared to the standard trials. Another explanation could be the parameters used to calculate the CBPT. For the calculation at sensor level, every single time point of the ERF was included in the test, whereas for the CBPT at source level, all time points were averaged. This could lead to a loss of statistical power.

The present source localization results show the main source activity in both centro-parietal and temporal regions of both hemispheres for target and standard stimuli. These results for the sources of the P3m are consistent with previous findings^19,39–42^. However, the sources of the modulation are not only found in the centro-parietal and temporal regions, but also in parts of the pre- and post-central gyrus and the inferior parietal lobe. All of these areas have previously been implicated in P3 sources before. For example, in a functional magnetic resonance imaging (fMRI) study, Bledowski and colleagues^43^ demonstrated P3b sources in the inferior parietal lobe, posterior parietal cortex and inferior temporal cortex in participants performing a difficult visual three-stimulus oddball paradigm. Furthermore, Halgren and colleagues^44^ identified superior parietal regions in intracranial recordings from epileptic patients as contributing to the P3b generation. McCarthy and colleagues^45^ reported activation of the inferior parietal lobe activation and middle frontal gyrus when the participants performed a visual task. In addition, Basile and colleagues^46^ showed, among other things, sources of the target P3 in the vicinity of the superior temporal sulcus (^46^, for a review see^47^).

Since, to our knowledge, the sources of the task difficulty-dependent P3m modulation have not been localized, it is difficult to relate our results to the literature. Our results suggest that the same generators are involved in the production of the P3m itself and also in the task-related difficulty modulation of the P3m. We were surprised to see that the source differences between easy and hard reached significance mainly within the left hemisphere. We can only speculate that this is due to the fact that we recruited only right-handed participants^48,49^. Considering the many different brain regions that contribute to the P3m, it is conceivable to assume that the source(s) of the task difficulty-dependent P3m modulation varies between modalities and tasks. Therefore, in future studies, it might be beneficial to localize the sources of an auditory as compared to a visual task in order to gain a better understanding of the interaction between the sources of the P3m generators. Perhaps also lesions in areas of the modulation generators are worth an investigation. Furthermore, investigating the specific generators in a distinct task could help to increase the stimulation effect in NIBS studies. For example, we used the sources localized in the present study and the established visual task for a follow-up study trying to amplify the amplitude of the P3 via tACS. We were able to increase the P3 amplitude after verum stimulation^25^ as compared to previous studies^50^.

In conclusion, we established a paradigm that reliably modulates the P3m amplitude and that can be utilized in future studies to investigate the underlying mechanisms of the P3m generation. Furthermore, the successful localization of the task difficulty dependent P3m modulation could pave the way for future research, such as intervention or lesion studies.

### Supplementary Information

Supplementary Figures.

## Data Availability

The complete raw MEG datasets are available in a public data repository (https://osf.io/n8kuz/).
