# Supplementary material for: Modulating the difficulty of a visual oddball-like task and P3m amplitude
Source: Sci Rep. 2024 Jan 17;14:1505. doi: 10.1038/s41598-023-50857-z (PMC10794184; doi:10.1038/s41598-023-50857-z)

Supplementary Material

**Supplementary Fig. 1** LCMV beamformer results of the target/standard effect.

Target/standard effect for the easy (left) and hard (right) condition. Similar to the findings of the P3m sources, the highest activity for the target/standard effect is located in centro-parietal regions for both easy and hard condition with smaller amplitudes in the hard condition.


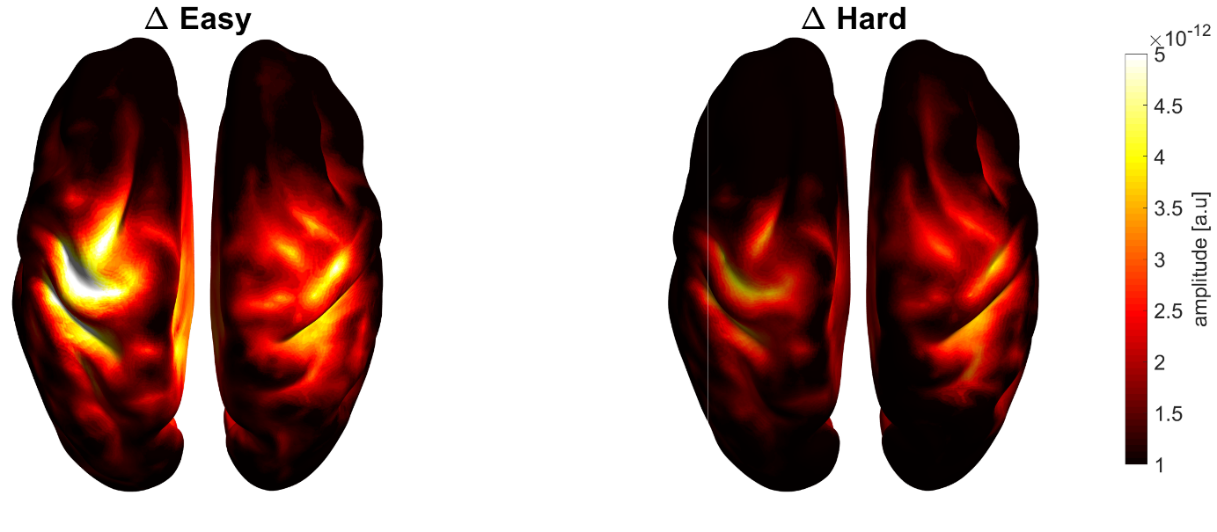


**Supplementary Fig. 2** Averaged results of the LCMV beamformer P1m, N1m and P3m for combined trials in each condition

a) Source activity of the P1m for combined stimuli for the easy (left) and hard (right) condition. Absolute values of source activity are depicted. The highest activity for the P1m is located in occipital regions. b) Source activity of the N1m for standard stimuli for the easy (left) and hard (right) condition. The highest activity for the N1m is located in the lateral occipital complex. c) Source activity of the P3m for standards for the easy (left) and hard (right) condition. The highest activity for the P3m is located in centro-parietal regions.


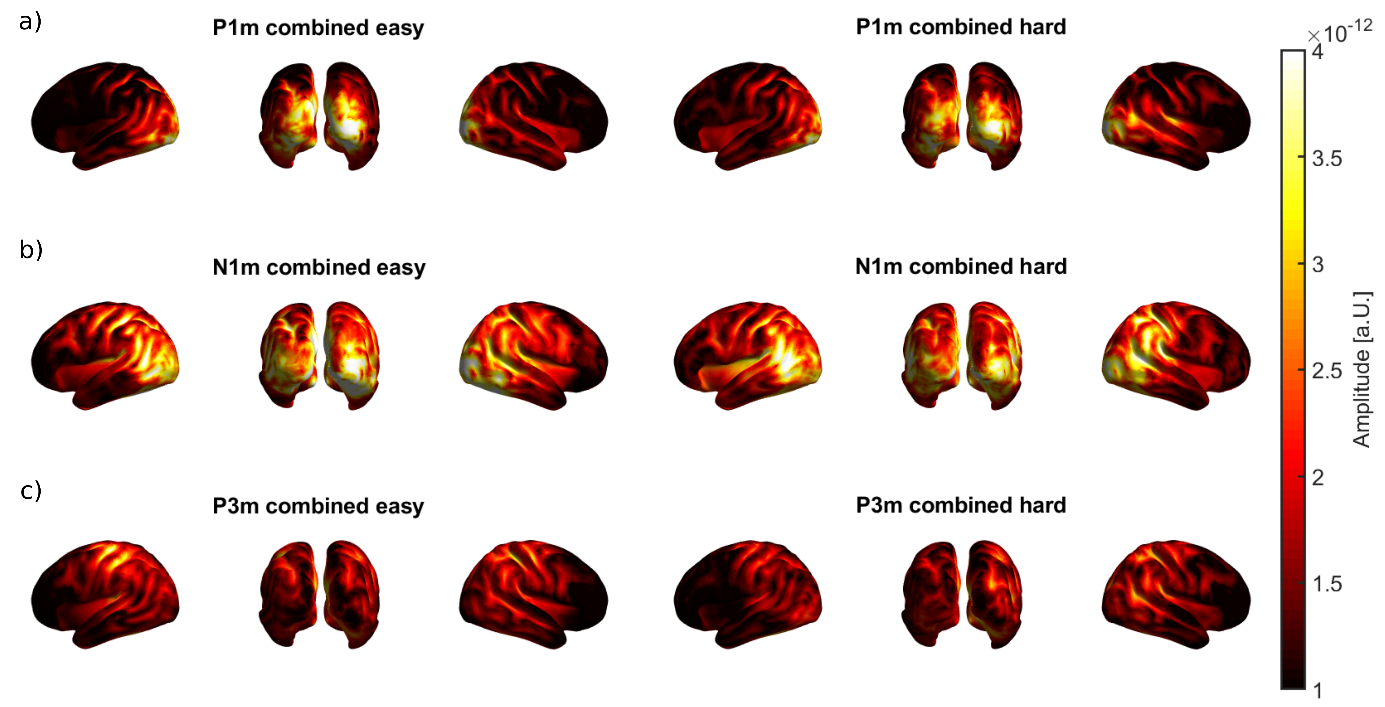


**Supplementary Fig. 3** Averaged results of the LCMV beamformer P1m, N1m and P3m for standards in each condition

a) Source activity of the P1m for targets stimuli for the easy (left) and hard (right) condition. Absolute values of source activity are depicted. The highest activity for the P1m is located in occipital regions. b) Source activity of the N1m for standard stimuli for the easy (left) and hard (right) condition. The highest activity for the N1m is located in the lateral occipital complex. c) Source activity of the P3m for standards for the easy (left) and hard (right) condition. The highest activity for the P3m is located in centro-parietal regions.


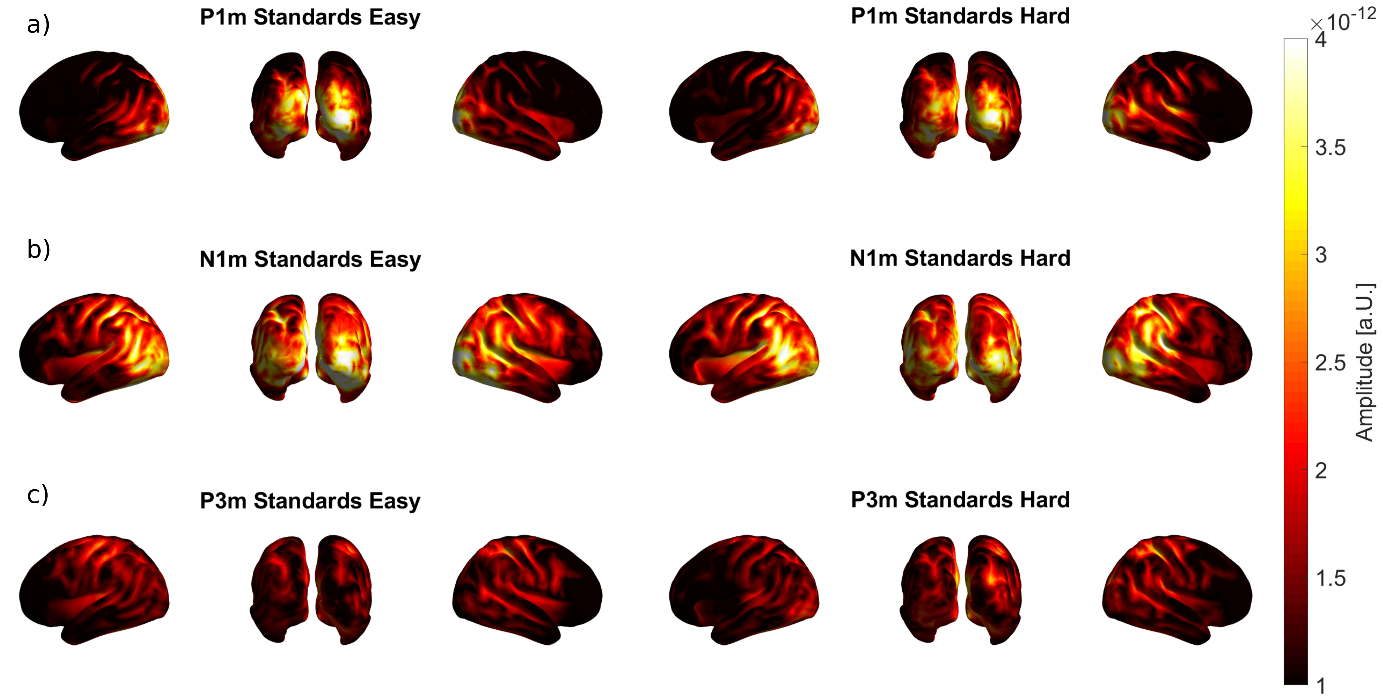


**Supplementary Fig. 4** Averaged results of the LCMV beamformer P1m, N1m and P3m for targets in each condition

a) Source activity of the P1m for targets stimuli for the easy (left) and hard (right) condition. Absolute values of source activity are depicted. The highest activity for the P1m is located in occipital regions. b) Source activity of the N1m for targets for the easy (left) and hard (right) condition. The highest activity for the N1m is located in the lateral occipital complex. c) Source activity of the P3m for target stimuli for the easy (left) and hard (right) condition. Absolute values of source activity are depicted. The highest activity for the P3m is located in centro-parietal regions.


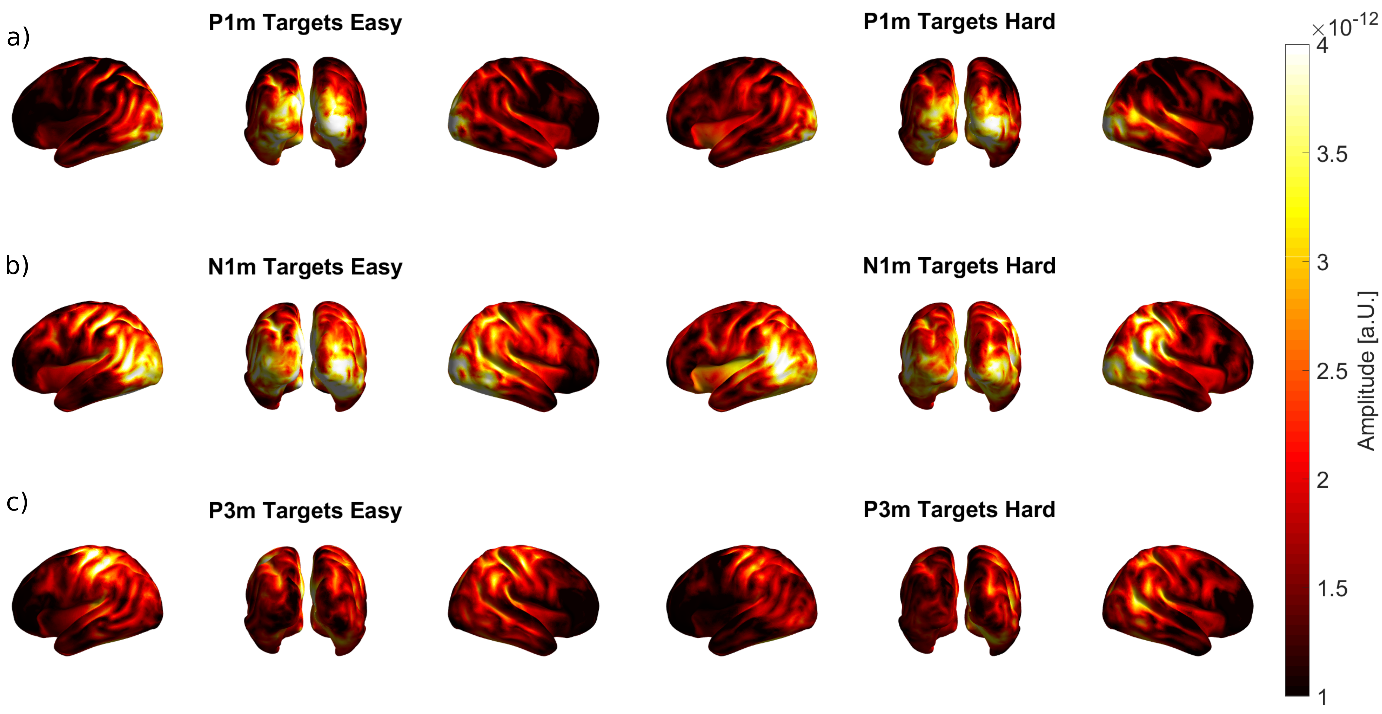

Supplement: Supplementary file 1 — Supplementary Figures. [file 41598_2023_50857_MOESM1_ESM.docx]
